# The Muscle Is Not a Passive Target in Myasthenia Gravis

**DOI:** 10.3389/fneur.2019.01343

**Published:** 2019-12-19

**Authors:** Jean-Thomas Vilquin, Alexandra Clarissa Bayer, Rozen Le Panse, Sonia Berrih-Aknin

**Affiliations:** Sorbonne Université, INSERM, Association Institut de Myologie (AIM), Paris, France

**Keywords:** myasthenia, muscle, neuromuscular junction, acetylcholine receptor, autoimmunity, cytokines, transcriptome

## Abstract

Myasthenia gravis (MG) is a rare autoimmune disease mediated by pathogenic antibodies (Ab) directed against components of the neuromuscular junction (NMJ), mainly the acetylcholine receptor (AChR). The etiological mechanisms are not totally elucidated, but they include a combination of genetic predisposition, triggering event(s), and hormonal components. MG disease is associated with defective immune regulation, chronic cell activation, inflammation, and the thymus is frequently abnormal. MG is characterized by muscle fatigability that is very invalidating and can be life-threatening when respiratory muscles are affected. MG is not cured, and symptomatic treatments with acetylcholinesterase inhibitors and immunosuppressors are life-long medications associated with severe side effects (especially glucocorticoids). While the muscle is the ultimate target of the autoimmune attack, its place and role are not thoroughly described, and this mini-review will focus on the cascade of pathophysiologic mechanisms taking place at the NMJ and its consequences on the muscle biology, function, and regeneration in myasthenic patients, at the histological, cellular, and molecular levels. The fine structure of the synaptic cleft is damaged by the Ab binding that is coupled to focal complement-dependent lysis in the case of MG with anti-AChR antibodies. Cellular and molecular reactions taking place in the muscle involve several cell types as well as soluble factors. Finally, the regenerative capacities of the MG muscle tissue may be altered. Altogether, the studies reported in this review demonstrate that the muscle is not a passive target in MG, but interacts dynamically with its environment in several ways, activating mechanisms of compensation that limit the pathogenic mechanisms of the autoantibodies.

## Introduction

Autoimmune Myasthenia Gravis (MG) is characterized by muscular weakness aggravated by exercise and improved by rest. The symptoms fluctuate, which makes the clinical diagnosis difficult. MG is mediated by antibodies (Ab) to components of the neuromuscular junction (NMJ), the muscle is thus the target of the autoimmune attack. About 85% of MG patients present Ab against the acetylcholine receptor (AChR) (1). In about 5% of MG patients, the autoreactive Ab target the muscle-specific kinase (MuSK) protein (2), which is involved in the clustering of AChRs (3). More recently, the agrin receptor LRP_4_ (low-density lipoprotein receptor-related protein 4), which forms a complex with MuSK, has been recognized as a novel autoantigen in a small proportion of MG patients without anti-AChR or -MuSK Ab (4). Antibodies to cortactin and agrin (5, 6) have been described, but their presence is most often concomitant to one of the other types of Ab.

MG is a complex disease to which genetic predispositions and defects of the immune system contribute (7–9). Thymic abnormalities are frequently found in the subgroup of MG with anti-AChR Ab but not in that with anti-MuSK Ab (10), and thymectomy has clinically favorable effects in AChR-MG (11), but not in MuSK-MG (12). MG patients with anti-AChR Ab can be classified in several subgroups according to the age of onset, the gender, thymic pathology, and anti-AChR antibodies [Reviewed in (13)]. While the defects of the immune system are richly described (7, 14, 15), reviews on the mechanisms taking place at the level of the muscle tissue are more sporadic (16–18), therefore we will focus on this aspect.

## Ultrastructural and Physiological Changes of the NMJ in MG

The development and maintenance of the NMJ are primarily dependent on the agrin-MuSK-LRP_4_ signaling system (19, 20). LRP_4_ and MuSK are anchored in the post-synaptic membrane. Agrin, secreted by the nerve terminal, binds to LRP_4_, which then binds to the extracellular domain of MuSK, resulting in phosphorylation and activation of MuSK (19). Phosphorylated MuSK recruits then Dok-7, an adaptor protein that becomes phosphorylated and recruits additional signaling molecules essential for synapse formation and AChR clustering (21).

**Figure 1:**
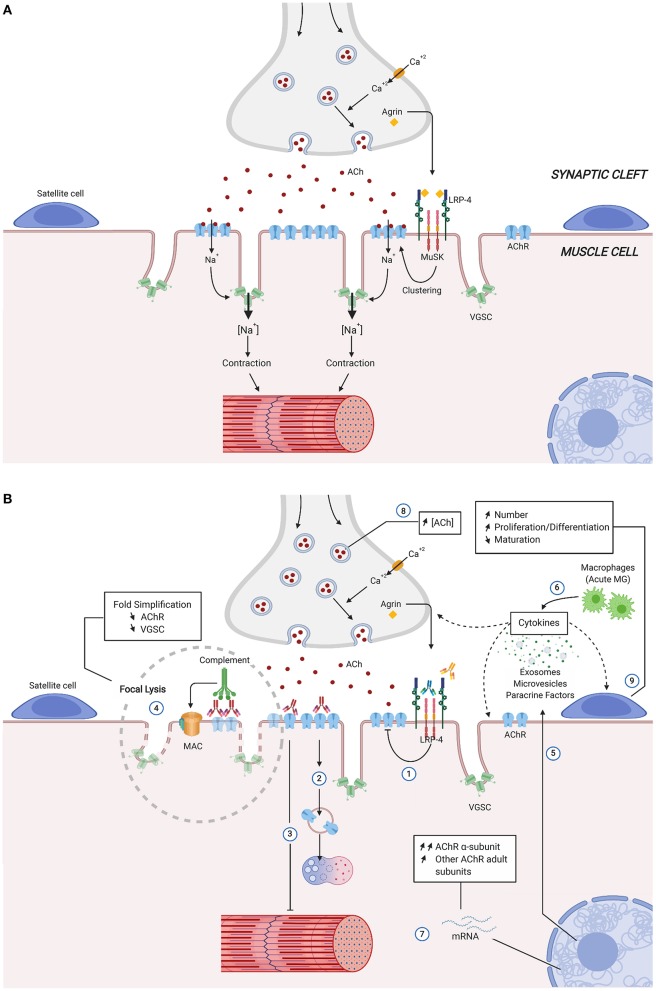
Simplified structure and function of the neuromuscular junction **(A)** and physiological Changes in autoimmune Myasthenia Gravis **(B)**. Direct, indirect consequences of the various autoantibodies and compensatory mechanisms, are identified by numbers. Anti-MuSK and anti-LRP_4_ autoantibodies act mainly by inhibiting AChR clustering (1). Anti-AChR antibodies reduce the expression of muscle AChR by removal of AChRs due to cross-linking, internalization, and degradation (2), functional AChR block (3), and activation of complement with formation of membrane-attack complexes that cause focal lysis (4). Blinding of anti-AChR antibodies also include muscle production of paracrine factors, microvesicles and exosomes, as well as cytokines (5) with potential effects over neighboring structures (satellite cells, muscle cells and nerve terminal). Pro-inflammatory environment can be enhanced during MG acute phase by infiltrating macrophages release of cytokines (6). Compensatory mechanism at molecular (7,8) and cellular levels (9) preserve MG muscle fibers from the AChR autoantibodies induced damage. Ach, Acetylcholine; AChR, Acetylcholine receptor; LRP-4, low-density lipoprotein receptor-related protein; MAC, membrane attack complex; MuSK, muscle specific kinase; VGSC, voltage-gated sodium channel.

Detailed structure and mechanism of the NMJ have been described in several reviews (22–25). Briefly, the post-synaptic membrane is characterized by deep junctional folds, the top of which are rich in AChRs, while voltage-gated Na+ channel (VGSCs) are concentrated in the depths [Review in (22, 24)]. There are ~10,000 AChR per square micrometer on the muscle surface in the motor plate, whereas the concentration is negligible outside the synaptic area. At the presynaptic side, 150,000–300,000 vesicles contain a quantum of acetylcholine (ACh) each (~10 000 molecules). Upon local depolarization, one quantal content (about 20 vesicles) is released in the synaptic cleft. The binding of ACh to AChRs induces an entry of Na+ into the muscle fibers, causing the local depolarization of the membrane and forming the endplate potential (EPP). The EPP stimulates the opening of the VGSCs, and upon reaching the firing threshold, a further influx of Na+ ions ensues, and the action potential spreads along the muscle fiber. It reaches and opens the stocks of intracellular calcium that finally trigger the muscle contraction (Figure 1A). In the healthy NMJ, the amplitude of EPP exceeds the threshold necessary to produce an action potential in the muscle. The ratio between the actual EPP and the threshold required to generate an action potential represents the safety factor of neuromuscular transmission, which is especially important during intense activation of the NMJ (26). In humans, the safety factor is about two, whereas it is higher in rodents or feline (27).

In AChR-MG disease, morphometric analysis reveals degenerative changes of the postsynaptic regions with widening and simplification of synaptic clefts and accumulation of debris in the synaptic zone (28, 29) (Figure 1B). In addition, nerve terminals are often smaller than normal size, and their sprouting may be observed (28). The degradation of the post-synaptic membrane results in a decrease in the expression of the AChR and the VGSCs channels, both contributing to the significant reduction of the safety factor: (1) EPP is lowered by the partial loss of functional AChRs and (2) the firing threshold is raised due to the reduction in the density of the sodium channels (30). During prolonged synaptic activity, as the quantal content of ACh normally runs down, the summation of EPP falls below the threshold, and they can no longer trigger the action potential of the muscle fibers (Figure 1B, numbers 1, 2, 3, 4). Then, several NMJ will present perithreshold EPP and intermittent transmission failures concomitantly, and the summation of several progressive blocks of NMJ transmission will lead to the MG symptoms (31).

Interestingly, the extraocular muscles (EOM) have physiologically less developed post-synaptic folding, hence a lower baseline safety factor, which could explain their high predisposition to dysfunction in MG (32). Furthermore, in ocular MG, these muscles are susceptible to complement-mediated attack due to a deficiency in complement-inhibitory proteins of the EOM and orbital tissue (33).

## Mechanisms of Action of the Ab

### Anti-AChR Ab

The pathogenicity of anti-AChR Ab has been shown by their ability to transfer the disease to control animals (34) and to reduce the number of α-bungarotoxin binding sites in myotube cultures (35). There is no correlation between the clinical severity of the disease and the Ab titer, but there is a correlation between the Ab titer and the ability of the sera to degrade AChR *in vitro* (36). However, in patients with immunosuppressive treatment, the changes in the level of anti-AChR antibodies is correlated with the clinical score (37).

Anti-AChR Ab can reduce the expression of muscle AChR by several mechanisms (Figure 1B): (1) removal of AChRs due to cross-linking and subsequent internalization (number 2); (2) functional AChR block (number 3), and (3) activation of complement with formation of membrane-attack complexes (MAC) that cause focal lysis (number 4) [Review in (38)]. Anti-AChR Ab are mainly IgG1 and IgG3 isotypes that bind the complement. This mechanism is likely the most pathogenic one: (a) there is an inverse relationship between the integrity of junctional folds and the abundance of C9, one molecule of the MAC (39); (b) mice mutated for complement factors (C3, C4, C5, C6) develop a lower incidence of MG upon active immunization, and their NMJ does not harbor the MAC [Review in (38)]; (c) Some patients with refractory MG have significant, often rapid, improvement in symptoms when treated with eculizumab, that inhibits the cleavage of C5 (40); (d) NMJ degradation decreases the safety factor and the efficacy of the transmission (41).

### Anti-MuSK Ab

As a receptor tyrosine kinase, MuSK interacts with a plethora of proteins and downstream pathways, some of which involved in nuclear anchoring, gene transcription, Wnt interactions, scaffolding, and AChR stabilization (20). MuSK-MG is often characterized by muscle atrophy and excellent response to plasma exchanges. Experimentally, animals that received repeated daily injections of patient IgG (42) or actively immunized with MuSK (43) show impaired neuromuscular transmission, with reductions in endplate AChR and EPP amplitudes [Review in (44)]. *In vitro*, anti-MuSK Ab induce inhibition of proliferation of a cell line, an effect correlated with disease severity and anti-MuSK Ab titer, that could explain the muscle atrophy in MuSK+ MG patients (45). The isotype of anti-MuSK Ab is generally IgG4 that lacks complement-activating properties and is considered functionally monovalent and is thus unable to induce antigenic modulation (46). Anti-MuSK Ab bind to a structural epitope in the first Ig-like domain of MuSK, prevent binding between MuSK and LRP_4_ and inhibit agrin-stimulated MuSK phosphorylation resulting in defects of AChR clustering (Figure 1B, number 1) (47). In addition, anti-MuSK Ab block binding of ColQ to the NMJ, that may lead to compromised agrin-mediated AChR clustering and AChR deficiency in MuSK-MG patients (48). Finally, some anti-MuSK Ab are directed against the Cysteine-rich domain of MuSK that mediates the Wnt-MuSK interactions (49). In summary, by contrast with anti-AChR Ab, anti-MuSK Ab induce a functional effect by interfering with MuSK signaling and AChR clustering.

### Anti-LRP_4_ Ab

Mice immunized with the extracellular domain of LRP_4_ exhibit MG-associated symptoms, including muscle weakness, reduced compound muscle action potentials, and compromised neuromuscular transmission (50, 51). Additionally, fragmented and distorted NMJs are evident at both the light and electron microscopic levels suggesting that LRP_4_ contributes to NMJ maintenance in adulthood. In nerve terminals, a reduction in synaptic vesicle density and ACh release is observed, while on the postsynaptic side, AChR density is significantly reduced, with flattened junctional folds (50). Interestingly, injection in mice of neural agrin (N-agrin) that binds to LRP_4_ leads to MG-associated symptoms, suggesting that agrin Ab may also play a role in MG pathogenesis (52).

## Molecular and Cellular Changes in MG Muscle

Several changes have been described inside and outside the giant syncytial muscle cell, and the importance of the local environment is increasingly considered (Figure 1B).

### Inflammation and Cytokines

It is generally admitted that diffuse signs of inflammation are not evident in the muscle of MG patients. First of all, immune cells are scarcely found (29) [Review in (53)]. Second, the transcriptome analysis did not reveal an inflammatory signature (54).

However, increased expression of cytokines (TNF-α, IL-1, and IL-6) due to infiltrating macrophages has been described in the muscle of models of experimental autoimmune MG (EAMG), during the early phase of the disease (55) (Figure 1B, number 6). In addition, muscle tissues can also produce immunologically relevant factors. Rat skeletal muscle exposed to anti-AChR Ab synthesizes MCP-1, IL-15, and NO, that promote the generation of disease symptoms (56–58) (Figure 1B, number 5). Besides, myotubes in MG and EAMG overexpress IP-10 and CXCR3, two molecules regulated by interferon-γ (59). Interestingly, the skeletal muscle also upregulates the PD-L1 in MG, which may participate in the control of the local immune-mediated damage through the function of a checkpoint inhibitor (60).

Some cytokines and inflammatory proteins are increased in the sera of MG patients and constitute an inflammatory environment (61–64), then direct effects of these molecules on muscles could be suspected. As a proof of concept, muscle cells are responsive to IL-4, IL-6, IFN-γ, and LPS, by producing immunologically relevant molecules and may become antigen-presenting cells (65, 66). The expression of Toll-like receptors by the skeletal muscle could favor the sensitization of the muscle to the environment [reviewed in (67)].

### Molecular Changes and Mechanisms of Compensation

Whether the molecular and cellular changes observed in and around the NMJ participate in the pathogenesis of MG disease or provide a mechanism of compensation are still an open question. Here, we will focus on two of these compensatory mechanisms.

First, the decreased expression in AChR is compensated by the release of an increased number of vesicles containing ACh, that has been shown in both muscles of MG patients and experimental rat models (Figure 1B, number 8) (27, 31, 68). The mechanism of this compensation may reside in several elements of the NMJ [Review in (27)]. At the presynaptic level, Ca^2+^/calmodulin-dependent protein kinase II (CaMKII) act through activation by Ca^2+^ (69), and this mechanism has been shown to be involved in the model of rats treated with alpha-bungarotoxin (70). Although not directly demonstrated in MG models, neuroligin (71), and Munc18 would act through the modulation of the number of docked release-ready vesicles (72). From the post-synaptic side, LRRK2 would trigger the increase of the size of the release-ready pool of vesicles (73). It has also been suggested that a specific pool of ACh vesicles, with a slower turn-over, would be used for transient increase of quantal content (74). LRP_4_ may be considered as a retrograde factor acting from muscle toward the presynaptic side (75). Clinically, the compensatory mechanism mediated by increased quantal content would be especially important during phases of intense, repetitive stimulation of the NMJ as it would counterbalance the natural rundown of quantal contents partially. Importantly, it should be noted that in MuSK-MG, this compensatory mechanism is not present, or it is blocked by the Ab, and these patients develop more severe disease (25, 27, 76).

Second, as a consequence of the attack of the AChR by Ab (Figure 1B, number 7), the degradation of the AChR is followed up by increased mRNA level expression of AChR subunits in muscles of myasthenic rats, rabbits, and mice compared with control animals (77–79). In MG patient muscle, the increase in AChR subunit transcripts correlates with the severity of the disease, indicating that this mechanism takes place only when the expression of AChR is significantly altered (80); *in vitro* studies show that the increase in AChR mRNA appears after a certain threshold loss of AChR (induced by monoclonal anti-AChR Ab) (80, 81). The expression of AChR is the resultant of loss and re-expression. Without such a mechanism of compensation, the AChR expression could be dramatically reduced, resulting in a fatal disease. Thus, this compensatory mechanism aims to balance the loss of AChR in human MG and is triggered above a certain degree of AChR loss (80).

Upregulation of AChR expression could also result from activation of neuregulin1/ErbB signaling pathway through overexpression of MuSK and rapsyn (82). Whether this pathway is implicated in MG has not been documented.

Other molecular alterations have been described in EAMG models and are likely to be secondary to the cross-reactive immune response. Notably, caveolin-3 shows aberrant overexpression. This muscle-specific membrane protein localized to the sarcolemma and T-tubule system is usually needed for muscle repair and skeletal muscle development (83). Also, the glucose-regulated protein 78 (GRP78) mRNA that is activated by ER stress is increased, suggesting that muscle weakness in MG might be caused by both NMJ disruption and ER stress (84). Another intriguing observation relates to the bone mineral density at skeletal sites, which is significantly decreased in the femur of EAMG mice compared to control animals, in parallel with the severity of the disease (85).

## Transcriptomic Analysis

A transcriptomics study was performed in 3 different muscles [EOM, diaphragm, and extensor digitorum longus (EDL)] in rats passively receiving anti-AChR Ab. Changes in 62 genes common among all muscle groups fall into four major categories (stress response, immune response, metabolism, and transcription factors). Interestingly, the EOM demonstrated a distinct RNA expression signature from EDL and diaphragm (86).

Transcriptome analyses were also performed on muscle biopsies from MG patients (compared with healthy controls) and on models of active EAMG in rats (compared with control rats). Similar changes in human and rat myasthenic muscles were found, highlighting the deregulation of genes included in the muscle fiber category. Also, genes related to cell metabolism and immune response were deregulated: Insulin-Like Growth Factor 1 (IGF-1) and Interleukin-6 (IL-6) pathways were identified. Indeed, increased IL-6 production was observed in human muscle cell cultures treated with MG sera or anti-AChR Ab. Besides, monoclonal anti-AChR Ab decrease Akt phosphorylation in response to insulin, indicating an effect of the Ab on cell metabolism (54). Since Akt plays a key role in multiple cellular processes such as growth and glucose metabolism, this reduced phosphorylation of Akt may have a significant impact on the muscle homeostasis, and fatigability observed in MG patients.

## Effects on Satellite Cells

Satellite cells (SCs) are quiescent muscular stem cells (Figure 1). After an injury, a process of muscle degeneration occurs, followed by the activation of the SCs that proliferate, become so-called myoblasts, differentiate, and fuse to give rise to new fibers (87).

Recently, the article by Attia et al. (88) unveiled an unexpected action of the anti-AChR Ab on these SCs. First, muscle sections from MG and EAMG contain an increased number of SCs identified by the Pax7 marker. Besides, SCs isolated from MG muscles proliferate as myoblasts and differentiate more actively than cells from control muscles. In addition, after a muscle injury induced in the EAMG mouse model, several changes were observed: a decrease in fiber size and MyoG mRNA expression and an increase in the number of fibers and embryonic myosin heavy-chain mRNA expression. These alterations suggest that as a result of the autoimmune attack, there is a delay in maturation of the muscle fibers.

A direct effect of the anti-AChR Ab on SC is unlikely since SCs do not express AChR. More likely, the binding of anti-AChR Ab to their antigens impairs the NMJ (see the mechanisms above) and alters the production of several paracrine factors, micro-vesicles, or exosomes by the muscle. These factors could then induce paracrine effects on the neighboring SCs associated with subtle modifications of the epigenetic signatures (Figure 1B, Number 9). This leads to the expression of MyoD and MyoG in MG SCs that will proliferate and differentiate more than in healthy ones.

Together, these data propose that MG muscles from EAMG mice regenerate worse than control ones. From a clinical perspective, symptom exacerbation upon sports practice or after a muscle injury could also be due to difficulties for MG patients to regenerate their muscle.

## Conclusion and Perspectives

In MG disease, the Ab to the different components of the NMJ have pathogenic consequences that are more extended than a focused effect on the target antigens. In other autoimmune diseases, the attack by the Ab and by the MAC would have induced the death of the target cells. In the case of the muscle, this does not occur, but activation of molecular transcription and signaling pathways, mechanisms of compensation, and biological effects on local cell types such as satellite cells demonstrate that the muscle responds actively. Thus, the muscle is not a passive target in MG but interacts dynamically with its environment in several ways. However, the number of studies examining theses processes is still quite limited. A better appraisal of these processes would allow identifying new mechanisms and pathways, and new levels for symptomatic medical interventions. New approaches are rapidly developing to model MG and facilitate such studies. Indeed, with the advent of pluripotent stem cells differentiation, and the growth of bioengineering, cocultures of human myogenic and neurogenic cells are possible in two (89) or three dimensions (90, 91), so as to study the effect of MG Ab, and/or to provide organoid-like platforms for the study of pathologies and their drug design.

## Author Contributions

SB-A and J-TV wrote the manuscript with support from RL. AB conceived and designed the figure.

### Conflict of Interest

The authors declare that the research was conducted in the absence of any commercial or financial relationships that could be construed as a potential conflict of interest.
